# A Cysteine Pair Controls Flavin Reduction by Extracellular Cytochromes during Anoxic/Oxic Environmental Transitions

**DOI:** 10.1128/mbio.02589-22

**Published:** 2023-01-16

**Authors:** Michael P. Norman, Marcus J. Edwards, Gaye F. White, Joshua A. J. Burton, Julea N. Butt, David J. Richardson, Ricardo O. Louro, Catarina M. Paquete, Thomas A. Clarke

**Affiliations:** a Babraham Institute, Babraham Research Campus, Babraham, Cambridge, United Kingdom; b School of Life Sciences, University of Essex, Colchester, United Kingdom; c School of Biological Sciences and School of Chemistry, University of East Anglia, Norwich, United Kingdom; d Instituto de Tecnologia Química e Biológica António Xavier, Universidade NOVA de Lisboa, Oeiras, Portugal; Duke University School of Medicine

**Keywords:** peroxide, flavoprotein, flavin, *Shewanella oneidensis*, cytochrome, cysteine

## Abstract

Many bacteria of the genus *Shewanella* are facultative anaerobes able to reduce a broad range of soluble and insoluble substrates, including Fe(III) mineral oxides. Under anoxic conditions, the bacterium Shewanella oneidensis MR-1 uses a porin-cytochrome complex (Mtr) to mediate extracellular electron transfer (EET) across the outer membrane to extracellular substrates. However, it is unclear how EET prevents generating harmful reactive oxygen species (ROS) when exposed to oxic environments. The Mtr complex is expressed under anoxic and oxygen-limited conditions and contains an extracellular MtrC subunit. This has a conserved CX_8_C motif that inhibits aerobic growth when removed. This inhibition is caused by an increase in ROS that kills the majority of S. oneidensis cells in culture. To better understand this effect, soluble MtrC isoforms with modified CX_8_C were isolated. These isoforms produced increased concentrations of H_2_O_2_ in the presence of flavin mononucleotide (FMN) and greatly increased the affinity between MtrC and FMN. X-ray crystallography revealed that the molecular structure of MtrC isoforms was largely unchanged, while small-angle X-ray scattering suggested that a change in flexibility was responsible for controlling FMN binding. Together, these results reveal that FMN reduction in S. oneidensis MR-1 is controlled by the redox-active disulfide on the cytochrome surface. In the presence of oxygen, the disulfide forms, lowering the affinity for FMN and decreasing the rate of peroxide formation. This cysteine pair consequently allows the cell to respond to changes in oxygen level and survive in a rapidly transitioning environment.

## INTRODUCTION

The facultative anaerobe Shewanella oneidensis MR-1 expresses a broad range of multiheme cytochromes that allow it to utilize a diverse range of substrates as terminal electron acceptors (1). These acceptors include soluble molecules such as dimethyl sulfoxide and nitrate and insoluble substrates such as Fe(III) and Mn(IV) oxides (2–4).

The central mechanism for electron transfer across the membrane involves the Mtr complex, a porin-cytochrome complex that consists of periplasmic decaheme MtrA, a large transmembrane pore MtrB, and the decaheme cytochrome MtrC on the surface of the cell (5). This Mtr complex forms an electron conduit through the outer membrane of *Shewanella* where MtrA directly exchanges electrons with the associated MtrC (6, 7). MtrC is extended approximately 80 Å above the lipid bilayer of the cell membrane, providing a surface for extracellular electron transfer to environmental substrates (7, 8).

While deletion of both MtrC and the analogous OmcA prevents the reduction of extracellular iron oxides, iron reduction by S. oneidensis is also dependent on the secretion of nanomolar concentrations of flavins (9, 10). These flavins can function as soluble shuttles, transporting electrons between extracellular cytochromes and the mineral surface. Reduction of certain iron oxides, such as ferrihydrite and lepidocrocite, by reduced flavin has been shown to occur at favorable rates (11). However, soluble reduced flavin mononucleotide (FMN) is a poor chemical reductant of mineral species such as hematite and goethite, and these minerals might be directly reduced by the extracellular cytochromes (12).

In addition to functioning as a soluble mediator, FMN has been shown to associate with MtrC and OmcA. *In vitro* studies of both oxidized MtrC and OmcA showed that these cytochromes were capable of interacting with FMN or riboflavin, although the dissociation constants obtained suggested that the interaction between MtrC and OmcA was likely to be transient, rather than stable (13). Theoretical binding sites have also been proposed on the surface of MtrC that are within electron transfer distance of adjacent heme groups (14–16).

MtrC is composed of four domains: an N-terminal β-barrel domain (domain I) is followed by a pentaheme domain (domain II), a second β-barrel domain (domain III), and a C-terminal pentaheme domain (domain IV). Domain III contains a conserved CX_8_C motif, which forms a disulfide bond between the two cysteines (17). Reduction of MtrC with glutathione caused a change in affinity for FMN, and cells expressing a plasmid-based *mtrC* with codons substituted to change the CX_8_C motif to AX_8_A were unable to grow aerobically. Disulfide bonds are often used as redox switches to regulate protein activity in different oxidative environments (18, 19), but it is unclear how the MtrC disulfide could be linked to both FMN association and the ability of S. oneidensis to respond to the presence of oxygen.

To investigate this, we analyzed the effects of site-directed mutagenesis on MtrC and show that removal of the disulfide causes global changes in the domain structure of MtrC that alter the affinity of MtrC for FMN. This modification produces cytotoxic levels of hydrogen peroxide under aerobic conditions while allowing mineral reduction under anoxic conditions. These results indicate that the MtrC disulfide acts as an oxygen sensor by disconnecting the EET chain to flavin in the presence of oxygen, thereby limiting formation of reactive oxygen that could otherwise damage the cell.

## RESULTS

### Synthesis of *MtrC* containing a modified CX_8_C motif restricts growth under aerobic conditions.

**FIG 1:**
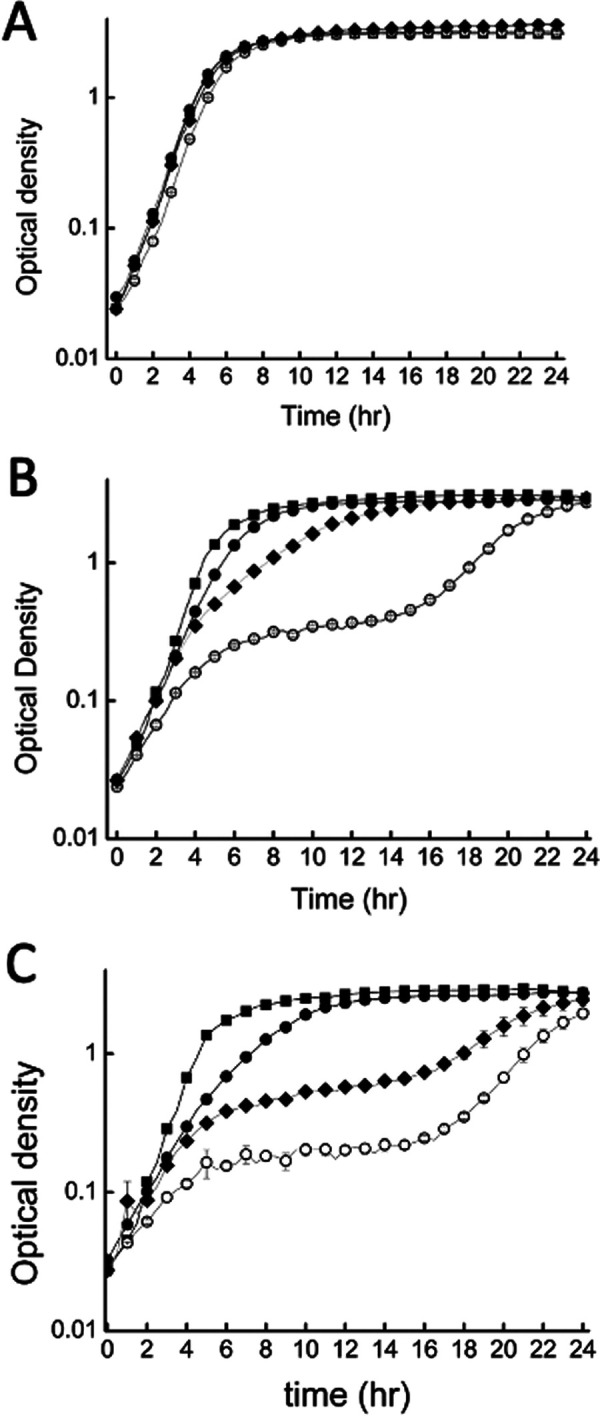
Semilog aerobic growth profiles of S. oneidensis pMtrC (squares), S. oneidensis pC444A (closed circles), S. oneidensis pC453A (open circles), and S. oneidensis pC444A, C453A (diamonds) induced with 0 mM arabinose (A), 5 mM arabinose (B), and 10 mM arabinose (C). All strains were grown aerobically in LB supplemented with 50 μg/mL of kanamycin at 30°C.

S. oneidensis *ΔmtrC* was transformed with the plasmid vectors shown in Table S1 in the supplemental material to make mutant S. oneidensis strains that would produce MtrC with modified CX_8_C motifs (Table 1). These strains were S. oneidensis pMtrC (CX_8_C), S. oneidensis pC444A (AX_8_C), S. oneidensis pC453A (CX_8_A), and S. oneidensis pC444A, C453A (AX_8_A). In the absence of the inducing agent, arabinose, all four strains showed similar growth under aerobic conditions (Fig. 1A). However, addition of 5 mM or 10 mM arabinose caused the S. oneidensis strains containing genes with a modified CX_8_C motif to exhibit biphasic growth (Fig. 1B and C). Initially, all strains enter a similar exponential phase for approximately 2 h, but then, growth of strains with a modified CX_8_C motif becomes arrested, with S. oneidensis pC453A being the most significantly affected. This continues for approximately 10 h before growth resumes and all strains reach the same final optical density (OD) after 24 h. Addition of arabinose to the same S. oneidensis strains did not affect the ability to grow under anaerobic conditions (Fig. S1), indicating that exposure to oxygen was responsible for the arrest in growth.

**TABLE 1:** S. oneidensis strains used in this study

Name	Host strain	Plasmid	Note(s)
S. oneidensis MR-1	S. oneidensis MR-1		
S. oneidensis ΔmtrC	S. oneidensis ΔmtrC		
S. oneidensis pMtrC	S. oneidensis ΔmtrC	pMtrC	CX_8_C motif
S. oneidensis pC453A	S. oneidensis ΔmtrC	pC453A	CX_8_A motif
S. oneidensis pC444A	S. oneidensis ΔmtrC	pC444A	AX_8_C motif
S. oneidensis pC444A, C453A	S. oneidensis ΔmtrC	pC444A, C453A	AX_8_A motif
S. oneidensis pMtrCsol	S. oneidensis ΔmtrC	pMtrCsol	Soluble form, CX_8_C motif
S. oneidensis pC453Asol	S. oneidensis ΔmtrC	pC453Asol	Soluble form, CX_8_A motif
S. oneidensis pC453Ssol	S. oneidensis ΔmtrC	pC453Ssol	Soluble form, CX_8_S motif

10.1128/mbio.02589-22.1 TABLE S1 Primers used to induce C444A and C453A mutations with deviations from wild-type *mtrC* highlighted. Download Table S1, DOCX file, 0.01 MB. Copyright © 2023 Norman et al. 2023 Norman et al. https://creativecommons.org/licenses/by/4.0/ This content is distributed under the terms of the Creative Commons Attribution 4.0 International license.

10.1128/mbio.02589-22.4 FIG S1 Anaerobic growth profiles of S. oneidensis *ΔmtrC* pMtrC (squares), S. oneidensis *ΔmtrC* pC444A (closed circles), S. oneidensis *ΔmtrC* p453A (open circles), and S. oneidensis *ΔmtrC* pC444A, C453A (diamonds) grown in LB supplemented with 50 μg/mL of kanamycin and 50 mM sodium fumarate at 30°C and induced with 0 mM arabinose (A), 5 mM arabinose (B), and 10 mM arabinose (C). S. oneidensis *ΔmtrC* pMtrC (open diamonds) grown in LB supplemented with 50 μg/mL of kanamycin, but no fumarate is shown in panel A. The average and standard deviation of 3 replicate experiments are shown. Download FIG S1, JPG file, 0.02 MB. Copyright © 2023 Norman et al. 2023 Norman et al. https://creativecommons.org/licenses/by/4.0/ This content is distributed under the terms of the Creative Commons Attribution 4.0 International license.

The relative levels of synthesis of each modified MtrC were measured. SDS-PAGE revealed that the presence of MtrC_C453A_ was significantly higher than either MtrC_C444A_ or MtrC_C444A,C453A_ (Fig. S2A) and was consistent with the more severe phenotype observed for S. oneidensis pC453A. Together, these data show that synthesis of MtrC with a modified CX_8_C motif limits cell growth under aerobic conditions. After 24 h of growth, the number of all MtrC isoforms in the membrane fractions was significantly less (Fig. S2B), indicating that expression of plasmid-based *mtrC* mutants had ceased.

10.1128/mbio.02589-22.5 FIG S2 Heme-stained SDS-PAGE gels of membrane fractions extracted from S. oneidensis strains grown for 2 h (A) and 24 h (B). Lane 1 in both gels is a molecular weight ladder of 250-, 150-, 100-, 75-, 50-, 37-, 25-, and 20-kDa markers. The arrows show the position of MtrC (75 kDa), Fcc3 (63 kDa), and MtrA (37 kDa), respectively. (A) Samples are shown with and without treatment with proteinase K (P-K). Lanes 2 and 3 S. oneidensis pMtrC; lanes 4 and 5, S. oneidensis pC444A; lanes 6 and 7, S. oneidensis pC453A; lanes 8 and 9, S. oneidensis pC444A, C453A; lane 10, S. oneidensis *ΔmtrC.* (B) Lane 2, S. oneidensis MR-1; lane 3, S. oneidensis pMtrC and S. oneidensis pC453A; lane 4, S. oneidensis pC444A; lane 5, S. oneidensis pC444A, C453A. Download FIG S2, JPG file, 0.02 MB. Copyright © 2023 Norman et al. 2023 Norman et al. https://creativecommons.org/licenses/by/4.0/ This content is distributed under the terms of the Creative Commons Attribution 4.0 International license.

MtrC_C453A_ was isolated using affinity chromatography as part of a stable Mtr complex, indicating that it could function as an electron acceptor for periplasmic MtrA and play a role in EET (Fig. S3A). To confirm that the MtrC_C453A_ was functional as an electron transport protein, the reduction rate of the Fe(III) mineral goethite was measured (Fig. S3B). The S. oneidensis *ΔmtrC* strain was unable to release Fe(II) from goethite, while the Fe(II) formation for both S. oneidensis pC453A and S. oneidensis pMtrC was the same, indicating that in these experiments, the role of MtrC in mineral reduction was not affected by substitution of Cys_453_.

10.1128/mbio.02589-22.6 FIG S3 (A) SDS-PAGE Coomassie-stained gel of purified MtrC_C453A-M_trAB complex in grayscale. (B) Reduction of goethite by S. oneidensis *ΔmtrC* (dotted line), S. oneidensis *ΔmtrC* pMtrC (straight line), and S. oneidensis *ΔmtrC* pC453A (dashed line). S. oneidensis strains were induced anaerobically and concentrated to an OD_600_ of 1.0 before addition to anaerobic LB media containing 5 mM arabinose and goethite at 20 mM Fe(III). One-milliliter samples were removed each hour, and soluble Fe(II) was measured using the ferrozine assay. Download FIG S3, JPG file, 0.02 MB. Copyright © 2023 Norman et al. 2023 Norman et al. https://creativecommons.org/licenses/by/4.0/ This content is distributed under the terms of the Creative Commons Attribution 4.0 International license.

### The formation of cytotoxic hydrogen peroxide is responsible for limiting S. oneidensis growth.

**FIG 2:**
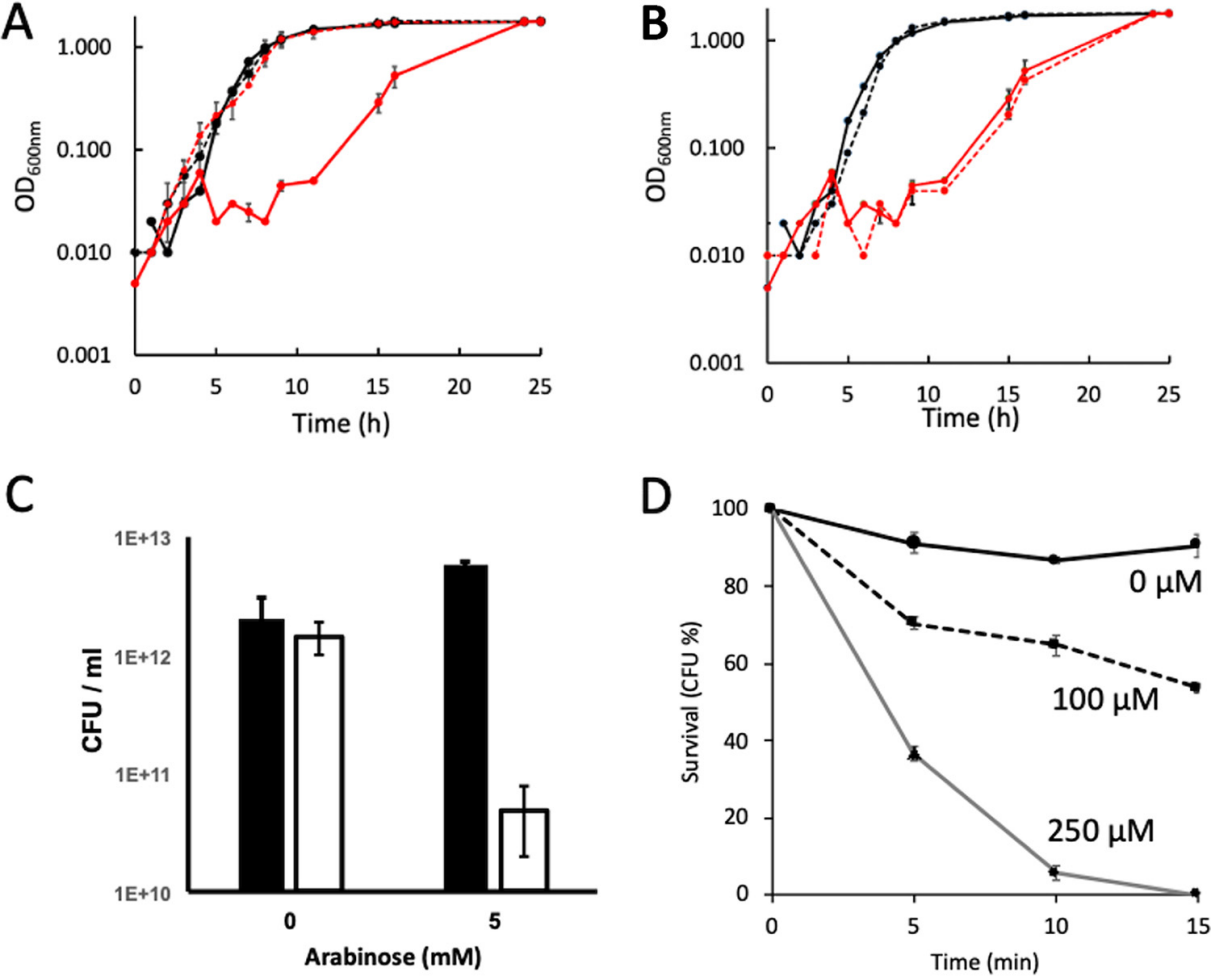
Effect of oxygen species on S. oneidensis cells in LB media supplemented with 5 mM arabinose and 50 μg/mL kanamycin. (A) Aerobic growth of S. oneidensis pMtrC (black lines) and S. oneidensis pC453A (red lines) in the presence (dashed lines) or absence (straight lines) of 0.3 U/mL of catalase. (B) Aerobic growth of S. oneidensis pMtrC (black lines) and S. oneidensis pC453A (red lines) in the presence (dashed lines) or absence (straight lines) of 0.3 U/mL of superoxide dismutase. (C) Cell viability after 3 h of induction with arabinose. Black bars represent S. oneidensis pMtrC; white bars indicate S. oneidensis pC453A. (D) Survival of S. oneidensis MR-1 in the presence of H_2_O_2_ as indicated. Error bars represent the standard error of four replicate experiments.

Cytotoxic effects of oxygen are typically linked to the reduction of oxygen to the reactive oxygen species (ROS), superoxide (O_2_^•−^), peroxide (O_2_^2−^), and hydroxyl radical (HO^•^). Peroxide is the most stable of these species and forms hydrogen peroxide (H_2_O_2_) under aqueous conditions at neutral pH. The formation of peroxide or superoxide by S. oneidensis pC453A was measured by adding the enzymes catalase or superoxide dismutase to aerobically growing cells (Fig. 2A and B). The addition of catalase allowed aerobic growth at a similar rate to S. oneidensis pMtrC, while the addition of superoxide dismutase had no effect on the aerobic growth profile. These results suggest that direct 2*e*^−^ reduction of oxygen to hydrogen peroxide, rather than superoxide, is responsible for the growth phenotype of the S. oneidensis pC453A mutant.

The concentration of hydrogen peroxide formed was measured by growing S. oneidensis strains overnight under anaerobic conditions using fumarate as an electron acceptor. The cultures were then exposed to air for 3 h, and the hydrogen peroxide concentration in the media was measured. The concentrations of hydrogen peroxide in air-exposed S. oneidensis pMtrC and S. oneidensis pC453A were 61 ± 36 μM and 257 ± 71 μM, respectively, revealing a 4-fold-higher concentration of hydrogen peroxide in the culture containing cells expressing MtrC_C453A_. S. oneidensis pMtrC and S. oneidensis pC453A grown aerobically for 24 h contained 39 ± 9 μM and 19 ± 3 μM hydrogen peroxide, respectively, indicating that hydrogen peroxide levels had become low enough to allow growth again, consistent with growth of S. oneidensis pC453A after the arrested growth phase.

The observed lag phase of cell cultures of S. oneidensis synthesizing MtrC_C453A_ (Fig. 2A and B) could be due to either the cells being alive but failing to divide or the cells actively dying in the presence of peroxide. To determine the number of viable cells, aerobic cultures of S. oneidensis pMtrC and S. oneidensis pC453A were sampled before and after induction with 5 mM arabinose (Fig. 2C). The number of cells in both cultures was approximately equal before induction; however, after 3 h, the number of viable S. oneidensis pC453A cells was 30 times lower than the starting number and over 100 times lower than the corresponding S. oneidensis pMtrC culture grown for the same length of time. This confirmed that induced synthesis of MtrC_C453A_ caused the death of S. oneidensis pC453A in the presence of oxygen, rather than suppression of cell division. Finally, to establish whether 250 μM H_2_O_2_ was cytocidal, cultures of S. oneidensis MR-1 cells were incubated with 100 or 250 μM H_2_O_2_ and spread onto plates (Fig. 2D). Exposure of S. oneidensis MR-1 to 250 μM H_2_O_2_ for 15 min was sufficient to kill 99% of cells, confirming that the elevated concentrations of H_2_O_2_ generated during aerobic growth of S. oneidensis pC453A were sufficiently high to cause the observed lag phase and subsequent cell death.

### Biochemical analysis of isolated MtrC C453A.

The *in vivo* data presented in Fig. 1 and 2 revealed that substituting Cys_453_ for alanine resulted in an S. oneidensis mutant that generated cytotoxic H_2_O_2_ under aerobic conditions, effectively turning a facultative anaerobe into a strict anaerobe that recovers by suppressing expression of the plasmid-based *mtrC*. The MtrC crystal structure showed that Cys_453_ forms a disulfide bond with Cys_444_ (17), but it is unclear how preventing the formation of this disulfide bond would increase the catalytic reduction of oxygen to H_2_O_2_. To resolve this, the soluble forms of both MtrC (MtrC_sol_) and MtrC C453A (MtrC_C453Asol_) were isolated. These constructs lacked the N-terminal LXXC lipid anchor motif and contained a C-terminal StrepTactin tag for ease of purification. The absorbance spectrum of isolated MtrC_C453Asol_ was identical to the absorbance profile of MtrC_sol_, consistent with all hemes having been correctly incorporated and no additional cofactors (Fig. S4A).

10.1128/mbio.02589-22.7 FIG S4 Normalized and overlaid UV-visible absorbance spectrum of MtrC_sol_ (red line) and MTRC_sol-C453A_ (black line) after gel filtration. (Inset) The region between 470 nm shows no difference, suggesting that no flavin was associated with either protein. Download FIG S4, JPG file, 0.02 MB. Copyright © 2023 Norman et al. 2023 Norman et al. https://creativecommons.org/licenses/by/4.0/ This content is distributed under the terms of the Creative Commons Attribution 4.0 International license.

**FIG 3:**
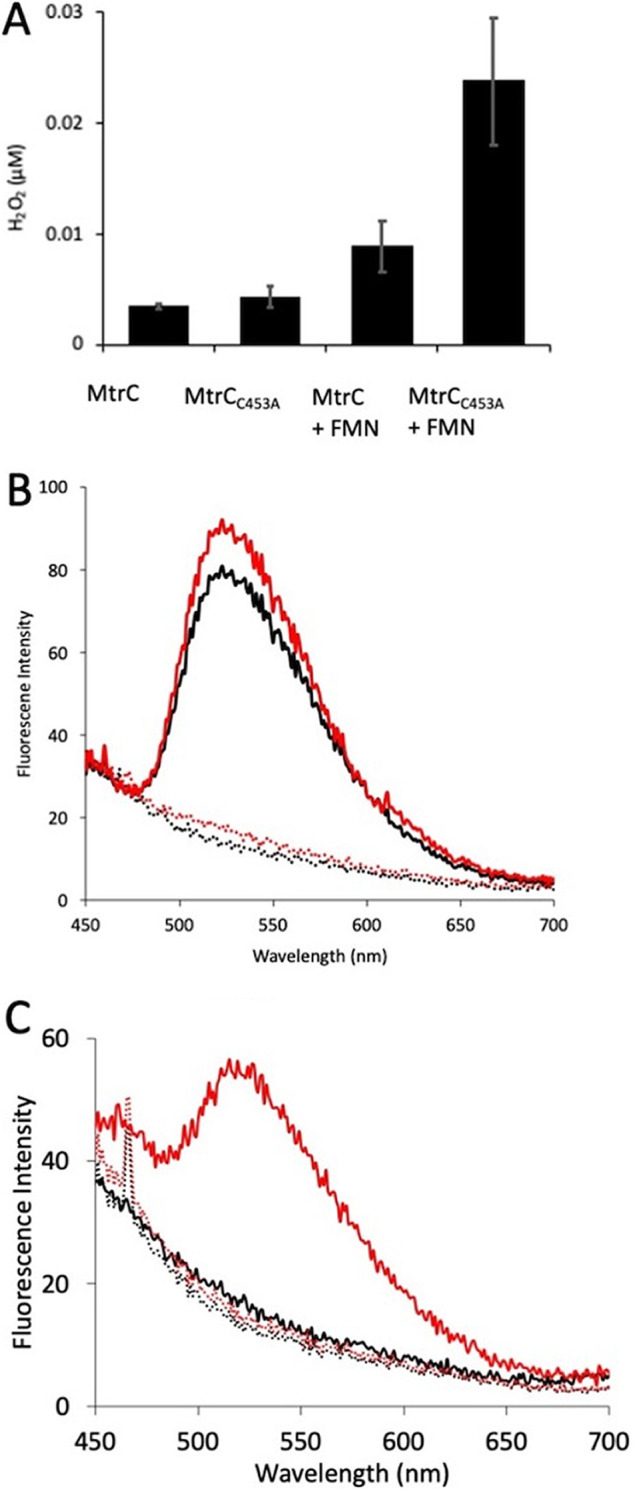
Interactions of FMN with MtrC_sol_ and MtrC_C453Asol_. (A) H_2_O_2_ generated by 0.5 μM reduced MtrC_sol_ or MtrC_C453A-sol_ in the presence or absence of 0.1 μM oxidized FMN after exposure to oxygen. (B) Fluorescence spectra of MtrC_sol_ (black) and MtrC_C453A-sol_ (red) samples incubated with 0 mM (dotted lines) or 1 mM DTT (solid lines) after elution through anaerobic PD10 size exclusion column under anaerobic conditions. Excitation held at 365 nm. (C) Fluorescence spectra of MtrC_sol_ (black) and MtrC_C453A-sol_ (red) samples incubated with 0 (dotted lines) or 50 μM sodium dithionite (solid lines) after elution through an anaerobic PD10 size exclusion column under anaerobic conditions.

To determine whether the reduced form of either MtrC_sol_ or MtrC_C453Asol_ could generate peroxide, 0.5-μM samples of each MtrC variant were reduced by titration with sodium dithionite under anaerobic conditions and then oxidized by exposure to air (Fig. 3A). The amount of hydrogen peroxide generated by both MtrC variants was similar, with a peroxide/electron efficiency of ~0.2%, assuming that 0.5 μM reduced MtrC contained 5 μM reduced hemes. This suggested that direct reduction of O_2_ by either MtrC was not the cause of the observed peroxide increase. These experiments were repeated in the presence of 0.1 μM FMN. For MtrC_sol,_ the presence of FMN doubled the amount of peroxide formed, while for MtrC_C453Asol_, there was a greater than 5-fold increase in the amount of peroxide formed, with a peroxide/electron efficiency of ~1%. These data reveal that FMN-catalyzed reduction of oxygen is the most likely reason for the observed increase in peroxide formation by cells producing MtrC_C453A_, most likely due to a change in the interaction between FMN and MtrC_C453Asol_. In previous studies, nuclear magnetic resonance (NMR) was used to measure a weak interaction between FMN and MtrC_sol_ (13). Here, this technique was used to determine the interaction between FMN and the MtrC variants MtrC_C453Asol_ and a variant MtrC with Cys_453_ substituted with serine (MtrC_C453Ssol_). The paramagnetic regions of the NMR spectra of MtrC_sol_, MtrC_C453Ssol_, and MtrC_C453Asol_ show similar signal positions and line widths (Fig. S5A), indicating that the structure and dynamics of these proteins are similar. The ^31^P NMR signal of FMN upon exposure to oxidized MtrC_C453Ssol_ was monitored, and the data revealed that binding occurs in the slow exchange regime in the NMR timescale, indicating strong binding (Fig. S5B). These results show that substitution of the cysteine increases the affinity of oxidized MtrC for FMN.

10.1128/mbio.02589-22.8 FIG S5 (A) ^1^H-1D NMR spectra of MtrC_sol_, MtrC_C453Ssol,_ and Mtr_C453Asol_ showing similar signals positions and linewidths. (B) ^31^P-1D NMR spectra of FMN in the presence of increasing amounts of MtrC_C453Ssol_, indicating strong binding. Stock solution of FMN was prepared in D_2_O (99.9%) in 20 mM potassium phosphate, pH 7.6, and 100 mM KCl. These NMR experiments were performed as previously described (13) with MtrC_C453Ssol_ in the same buffer. Binding of FMN to MtrC_C453S_ was monitored by ^31^P NMR spectroscopy in a Bruker Avance 500 II+ spectrometer equipped with a P-SEX probe at 25°C. ^31^P experiments were run with proton decoupling, processed with exponential apodization, and analyzed in TopSpin 3.2. Download FIG S5, JPG file, 0.02 MB. Copyright © 2023 Norman et al. 2023 Norman et al. https://creativecommons.org/licenses/by/4.0/ This content is distributed under the terms of the Creative Commons Attribution 4.0 International license.

MtrC_sol_ can form an isolatable complex with FMN under anaerobic conditions in the presence of a thiol-reducing agent (17). The ability of MtrC_C453Asol_ to form a similar complex was investigated by incubating MtrC_sol_ or MtrC_C453Asol_ with an excess of FMN and 1 mM dithiothreitol (DTT) under anaerobic conditions for 30 min before passage through a desalting column and fluorometric analysis (Fig. 3B). The fluorescent spectra of both MtrC were similar, showing that MtrC_C453Asol_ would form a similar FMN complex to MtrC_sol_ in the presence of DTT. DTT is typically used as a disulfide-reducing agent, and so, it was assumed that the role of DTT was to reduce the Cys_444_-Cys_453_ disulfide, and this formed the MtrC-FMN complex. Substitution of Cys_453_ with alanine would prevent the disulfide from forming and remove the requirement of DTT for complex formation. However, when MtrC_C453Asol_ was incubated with FMN in the absence of DTT, the purified fluorescent spectra of MtrC_C453Asol_ were identical to MtrC_sol_, showing that FMN had not bound (Fig. 3B). DTT is capable of reducing both FMN and heme, and it was possible that the interaction between MtrC and FMN also required the specific reduction of either MtrC and FMN. This was explored by incubating MtrC_C453Asol_ or MtrC_sol_ with FMN and 50 μM sodium dithionite. The fluorescence spectrum of the isolated complexes revealed copurification of FMN with MtrC_C453Asol_ but not MtrC_sol_ (Fig. 3C), consistent with the reduced forms of FMN and MtrC heme facilitating an increase in the affinity of FMN for MtrC_C453Asol_. These data suggest that the interaction between FMN and MtrC is determined by the redox state of MtrC/FMN as well as the CX_8_C motif.

### Structural comparison of MtrC_sol_ and MtrC_C453Asol_.

Biochemical analyses revealed that the CX_8_C motif has an important role in controlling interactions between FMN and MtrC. When the disulfide bond is broken, either through reduction or mutagenesis, MtrC is more able to bind FMN, suggesting a conformational change that would affect the affinity of MtrC for FMN.

**FIG 4:**
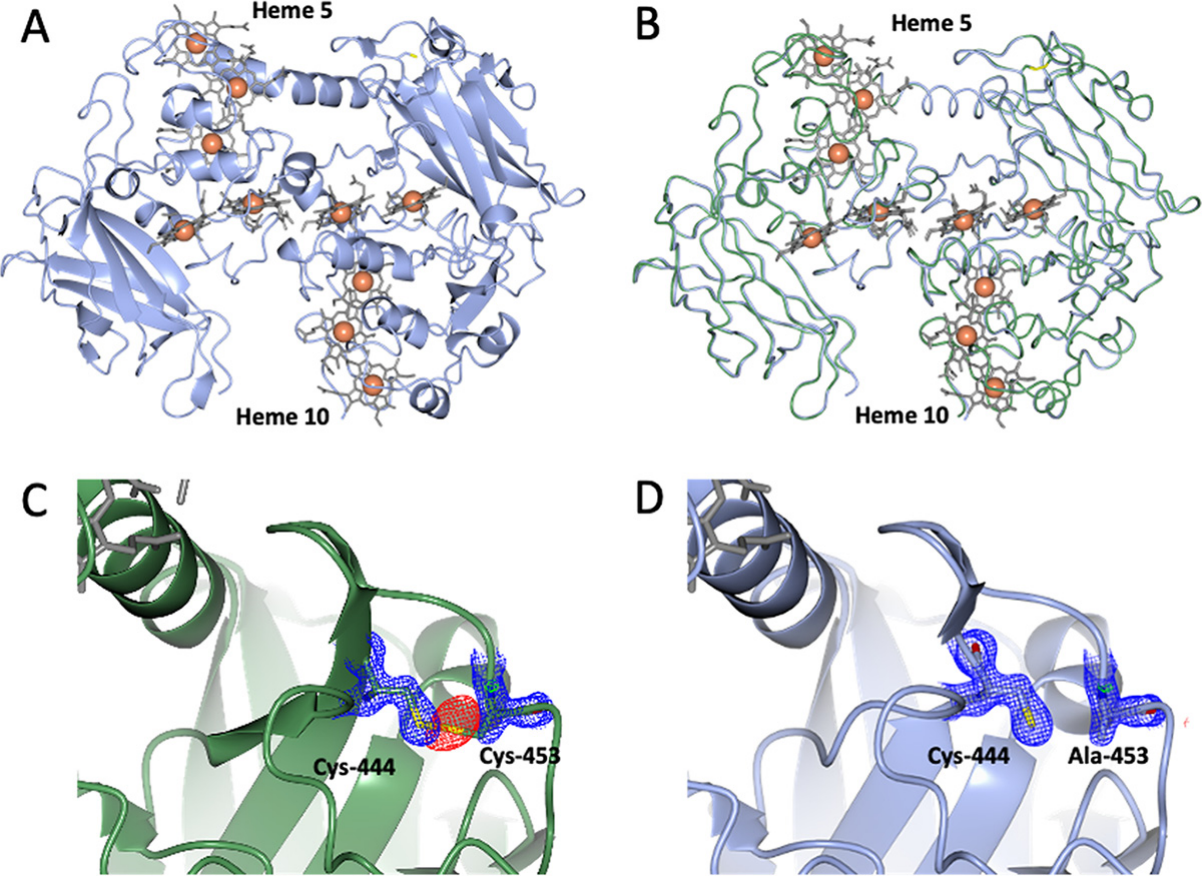
Crystal structure of MtrC_C453sol_ (PDB accession no. 7QTH). (A) Cartoon representation of MtrC_C453sol_. Hemes are shown as gray cylinders with the iron atoms represented as orange spheres. Cys-444 and Ala-453 side chains are shown as cylinders. (B) Structural superposition of the structure of MtrC_C453sol_ (blue) with that of MtrC_sol_ (green; PDB accession no. 4LM8). (C and D). 2F_o_-F_c_ (blue) and F_o_-F_c_ (green/red) electron density maps, contoured at 1.2 and 3 sigma, respectively, resulting from refinement of coordinates for MtrC_sol_ (C) and MtrC_C453sol_ (D) against the MtrC_C453sol_ data. For clarity, solvent molecules are not shown, and maps are clipped to within 2 Å of residues 444 and 453.

MtrC_C453Asol_ was crystallized, and a structural model was determined to a resolution of 1.9 Å (Table S3). The overall structure was almost identical to the wild-type structure with a root mean square displacement (RMSD) of 0.297 Å (Fig. 4A and B). The area around Cys_453_ was largely conserved, although F_o_-F_c_ maps revealed a negative patch of density where the disulfide was formed between Cys_453_ and Cys_444_ in MtrC_sol_ (Fig. 4C). This confirmed that Cys_453_ was replaced with alanine and the disulfide was missing, but there were no other significant changes in the environment near residue 453. In addition, there was no electron density consistent with the covalent attachment of anything to Cys_444_ (Fig. 4C and D). These data show that the Cys_453_ substitution caused little perturbation of MtrC beyond the removal of the disulfide within the second β-barrel domain of MtrC. This surprising result did not explain how the removal of a disulfide bond could control binding and reduction of FMN.

**FIG 5:**
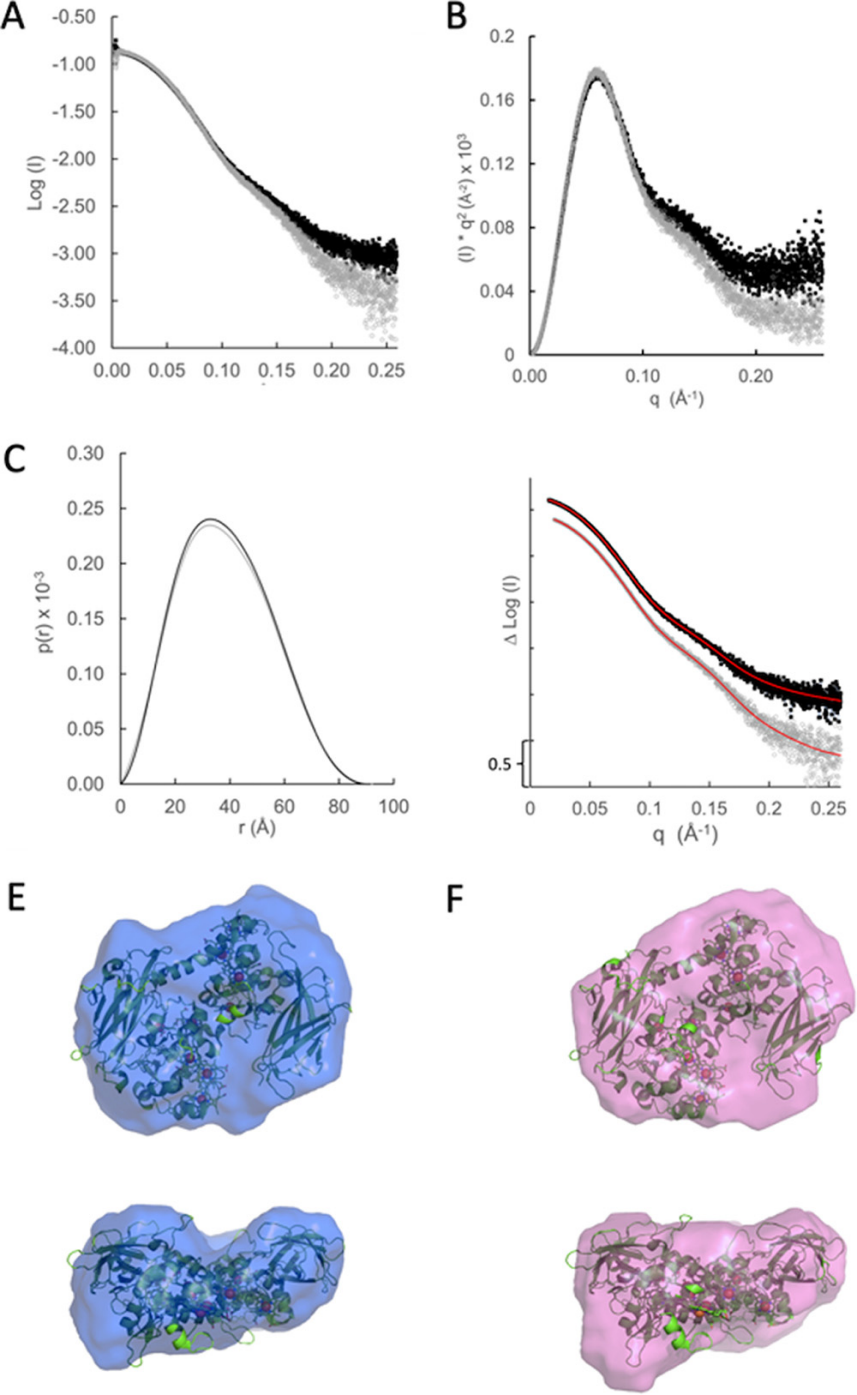
Small-angle X-ray scattering analysis of soluble MtrC (black) and MtrC_C453A_ (gray). (A) Average scattering curves for MtrC_sol_ and MtrC_C453Asol_ showing a divergence at *q* values greater than 0.1. (B) Scattering data of MtrC_sol_ and MtrC_C453Asol_ plotted as (*I*)×*q*^2^ versus *q* (Kratky plot). (C) *P*(*r*) distance distribution plot showing the probability distribution of both MtrC_sol_ and MtrC_C453Asol._ (D) Molecular envelopes were generated from the scattering data using DAMMIN. The simulated curves for all data are shown in red. The DAMMIN curves fitted to the scattering data with chi-square values of 1.133 and 1.124 for MtrC_sol_ and MtrC_C453Asol,_ respectively. (E, F) The molecular envelopes of MtrC_C453Asol_ (E) and MtrC_sol_ (F) generated by DAMMIN were fitted to the MtrC crystal structure (PDB accession no. 4LM8). The NSD values between the MtrC crystal structure and MtrC_C453Asol_ or MtrC_sol_ were 1.28 and 1.17, respectively.

One possible explanation for the discrepancy between the catalytic and structural differences could be that the crystal structure represents a stable conformation of the two MtrC isoforms and that a change in the conformational flexibility of MtrC causes the change in FMN interaction upon disulfide reduction. To investigate this further, samples of MtrC_sol_ and MtrC_C453Asol_ were analyzed using size exclusion chromatography combined with small-angle X-ray scattering (SEC-SAXS). Both samples eluted at the same positions and gave a uniform radius of gyration (*R_g_*) across the peak (Fig. S6). Scaling and signal averaging of both samples gave scattering curves showing a high degree of similarity at low scattering factors (*q*), but as *q* increased, the two scattering curves deviated, suggesting a subtle difference between the two conformations (Fig. 5A).

10.1128/mbio.02589-22.9 FIG S6 SEC-SAXS signal plots. Samples of 7.5 mg/mL MtrC_sol_ (A) and MtrC_C453Asol_ (B) were eluted through a Shodex KW402.5-4F column on Beamline B21 at Diamond Light Source. Red diamonds show the integrated area of the ratio of the sample SAXS curve to the estimated background for each scan, while the open circles are the Guinier *R_g_* values calculated for the corresponding scan. Log_10_ intensity plots of subtracted and merged SAXS frames for MtrC_Sol_ (C) and MtrC_C453Asol_ (D). Black represents averaged buffer frames subtracted from averaged sampled frames. Cyan represents median of the buffer frames subtracted from the averaged sample frames. Poor buffer subtraction would cause a displacement between the two curves at high *q.* Download FIG S6, JPG file, 0.02 MB. Copyright © 2023 Norman et al. 2023 Norman et al. https://creativecommons.org/licenses/by/4.0/ This content is distributed under the terms of the Creative Commons Attribution 4.0 International license.

Plotting the data as a function of *I*×*q*^2^ versus q (Kratky plot [Fig. 5B]) revealed profiles for both complexes consistent with folded proteins. Both samples were homogenous at low *q,* but the slight increase in scattering at higher *q* suggested that MtrC_sol_ was more flexible than MtrC_C453Asol_. Distance distribution [*P*(*r*)] curves provided *R_g_* and maximum dimension (*D*_max_) values of 29.41± 0.14 and 91.2 Å for Mtr_sol_ and similar *R_g_* and *D*_max_ values of 29.44 ± 0.14 and 91.5 for MtrC_C453A-sol_ (Fig. 5C), indicating that both structures were very similar. The *P*(*r*) curves were finally used to generate structural models for both complexes using DAMMIN (20). These molecular envelopes fitted well to the data, with chi-square values of 1.133 and 1.124 for MtrC_sol_ and MtrC_C453Asol_, respectively (Fig. 5D). The molecular envelopes were then fitted to the MtrC crystal structure (Fig. 5E and F). Surprisingly, neither envelope matched the crystal structure. Normalized spatial discrepancy (NSD) values between the MtrC model and the molecular envelopes were 1.17 (MtrC_C453Asol_) and 1.28 (MtrC_sol_), suggesting that, while neither solution model fitted the structure perfectly, MtrC_C453Asol_ in solution more closely resembles the crystal structure than MtrC_sol_. These differences suggest solution-based transient conformations that may explain why MtrC_C453Asol_ and MtrC_sol_ show different affinities for FMN.

## DISCUSSION

S. oneidensis MR-1 is known to encode over 40 different cytochromes and has a high concentration of intracellular iron (1, 21). In comparison to other bacteria, S. oneidensis is susceptible to damage from ionizing radiation. This susceptibility is not a consequence of direct radiation damage but indirect through the activation of several reactive species, notably ROS. It is most likely that the susceptibility of S. oneidensis MR-1 to ROS occurs due to the single-electron reduction of peroxide to hydroxyl radicals through the Fenton reaction catalyzed by Fe^2+^ available inside the cell (21). Under saturating oxygen conditions, *mtr* expression is likely to be suppressed; however, as the cell population increases, the oxygen concentration decreases and the expression of the *mtr* operon begins. For S. oneidensis pC453A, this would cause the formation of peroxide. This could explain the initial exponential growth phase that stops at an OD between 0.1 and 0.3.

To understand how the CX_8_C motif in MtrC can protect against oxidative damage, a range of plasmids containing mutated *mtrC* genes were constructed and inserted into an *mtrC* knockout strain of S. oneidensis. The growth of MtrC variants unable to form the Cys_444_-Cys_453_ disulfide bond under aerobic conditions was severely impaired compared with the wild-type organism. The biphasic growth could be explained by the accumulation of hydrogen peroxide, reaching cytotoxic levels after 2 h and preventing further growth. After approximately 10 h, there was a second, recovery exponential phase once the bacteria no longer expressed recombinant MtrC. The best-expressed recombinant MtrC variant, MtrC_C453A_, was as effective a goethite reductase as wild-type MtrC under the conditions tested, showing that the disulfide bond is not required for anaerobic mineral respiration.

*In vitro* analysis of soluble MtrC isoforms revealed that FMN reduction was responsible for the increased peroxide formed by MtrC_C453Asol_ and the interaction between FMN and MtrC was controlled by both the redox state of the Cys_444_-Cys_453_ pair and the redox state of FMN-MtrC. No changes were detected in the crystal structure of MtrC_C453Asol_, suggesting that the effect of removing the disulfide caused changes to the stability of MtrC, as indicated by SAXS experiments that showed differences in intensity at higher scattering angles. The structure of *Shewanella* MtrC is unusual in containing two multiheme domains (domains II and IV) separated by beta-barrel domain III containing the CX_8_C motif. It would be possible to disrupt electron transfer between the two multiheme domains through movement of the region connecting domain II to domain III, close to the CX_8_C motif. Molecular dynamic docking simulations indicated flavin was most likely to associate near heme 2 of domain II (15); however, it is not clear how this binding site would be affected by the redox state of the CX_8_C motif some 30 Å away.

These results help to clarify the reason for the conserved disulfide bond on the surface of MtrC cytochromes. Under anaerobic mineral-respiring conditions, the MtrC disulfide can be reduced, increasing the association of secreted FMNs to the cytochrome and allowing effective reduction of FMN that can then reduce insoluble metal oxides. If the cell is exposed to oxygen, the two cysteines become oxidized and form a disulfide bond, which causes discrete conformational changes in MtrC and limits the association and reduction of FMN, which, in turn, prevents the FMN-mediated production of ROS. In our MtrC_C453A_ isoform, the formation of the disulfide bond is prevented, trapping MtrC_C453A_ into a state that favors FMN reduction under aerobic conditions, leading to increased production of peroxide and cell damage. The disulfide of MtrC therefore functions as a switch, changing the reactivity of MtrC on exposure to oxygen.

*Shewanella* species live in the waters and sediments of lakes, rivers, and oceans and consequently may experience rapid changes in the oxygen concentration of the immediate environment. Anaerobic expression of *mtrCAB* by S. oneidensis MR-1 allows the continuous flux of electrons generated inside the cell to access the surface of the cell, where they can be transferred to extracellular electron acceptors. Under aerobic conditions, these electrons would reduce oxygen, generating the ROS that then attack the cell. To minimize the risk of cellular damage, S. oneidensis exposed to oxygen must immediately decrease the rate of ROS production to concentrations that can be dealt with by the diheme peroxidases present in the periplasm. This surface-exposed CX_8_C motif provides a rapid switching mechanism to prevent FMN reduction and allows *Shewanella*-containing MtrCAB to prevent the formation of ROS under aerobic conditions.

## MATERIALS AND METHODS

### Construction of strains.

PCR was performed using primers listed in Table S1 in the supplemental material. For generation of cysteine mutants in the membrane-bound form of MtrC, plasmid pMtrC, a pBAD202/d-TOPO cloning vector containing the *mtrC* gene from S. oneidensis MR-1, was used (22). For generation of cysteine mutants in the soluble form of MtrC, a modified *mtrC* plasmid was used in which the nucleotide sequence encoding the lipid anchor site and signal peptide of MtrC was replaced with the signal peptide sequence of MtrB (pMtrCsol). A nucleotide sequence encoding a StrepTactin tag was inserted at the 3′ end of *mtrC* to give a C-terminal affinity tag.

Single-cysteine mutant plasmids served as the templates in further PCRs to generate double mutant *mtrC* where both cysteine residues were substituted for alanine. Plasmids are summarized in Table S2.

10.1128/mbio.02589-22.2 TABLE S2 Summary table of plasmids generated through site-directed mutagenesis. Plasmid name, expression vector, purification tag, and, if membrane anchor is present, are shown. Download Table S2, DOCX file, 0.01 MB. Copyright © 2023 Norman et al. 2023 Norman et al. https://creativecommons.org/licenses/by/4.0/ This content is distributed under the terms of the Creative Commons Attribution 4.0 International license.

Either pMtrC, pC444A, pC453A, or pC444A, C453A was transformed into S. oneidensis *ΔmtrC* (LS661). The final strains generated are described and summarized in Table 1. S. oneidensis *ΔmtrC* (LS661) cell lines containing plasmids will be referred to as S. oneidensis pMtrC (native MtrC expressing), S. oneidensis pC444A (single-cysteine substitution at amino acid position 444), S. oneidensis pC453A (single-cysteine substitution at amino acid position 453), or S. oneidensis pC444A, C453A (double-cysteine substitution).

### Growth of S. oneidensis strains.

For aerobic growth curves, S. oneidensis strains were inoculated into LB cultures with 50 μg/mL kanamycin and 0 to 10 mM arabinose in a 96-well plate. This was incubated aerobically at 30°C in a FLUOstar Omega plate reader (BMG Labtech) under agitation. The OD_600_ was recorded at 30-minute intervals.

For anaerobic growth curves, LB medium was supplemented with 50 mM sodium fumarate to act as an electron acceptor in place of O_2_. One-milliliter aliquots of cultures were transferred into a 48-well plate and transferred into an anaerobic glove box and left for 30 min. A coverslip was adhered before the plate lid was glued into place using an airtight adhesive. The plate was then removed from the glove box and incubated as described above but without agitation. A control well containing LB lacking sodium fumarate and inoculated with S. oneidensis MR-1 was used to confirm anaerobic conditions.

### Indigo carmine assay for H_2_O_2_ measurements.

S. oneidensis pMtrC and S. oneidensis pC453A were grown anaerobically in LB media with 50 mM sodium fumarate and 5 mM arabinose. Samples were removed before the cultures were supplemented with fresh LB media and grown aerobically for 2 h, and further samples were taken. Samples were taken and assayed for H_2_O_2_ using Oxygen Vacu-Vials kit K-7503 (CHEMetrics) after sparging with argon. Final samples were assayed after incubation with 0.3 U catalase and further sparging with argon to remove oxygen formed from the process. The resulting reactions were analyzed at 610 nm with the absorbance of sparged samples before and after catalase incubation attributed to hydrogen peroxide.

### Protein expression and purification.

LB medium supplemented with 50 μg/mL of kanamycin and 20 mM sodium fumarate was sparged with nitrogen and inoculated with S. oneidensis strains. Cultures were incubated at 30°C with shaking, and cell growth was monitored. When OD_600_ had reached ~0.4, 5 mM arabinose was added to cultures, and oxygen was introduced to cultures before again being incubated at 30°C shaking at 220 rpm for a further 2 h. Afterward, cells were centrifuged and resuspended in 20 mM HEPES, pH 7.8, to an OD_600_ value of 0.5. Samples were analyzed using SDS-PAGE by the heme stain method to assess cytochrome expression levels.

### Protein purification.

S. oneidensis strains containing either pC453Asol, pMtrCsol, or pC453S were inoculated into LB media supplemented with 50 μg/mL kanamycin and 0.3 U/mL catalase and incubated at 30°C. When OD_600_ reached ~0.5, cultures were induced with 5 mM arabinose and incubated aerobically at 30°C for 10 h before centrifugal harvesting. Soluble MtrC isoforms were isolated as described previously (17). The detergent-solubilized MtrC_C453A_AB complex was isolated using a different method as described previously (23). Fractions were analyzed via SDS-PAGE using Coomassie and heme staining.

### Crystallographic analysis of MtrC_C453Asol_.

Mtr_C453Asol_ samples were concentrated to 10 mg/mL before crystallization using sitting-drop vapor diffusion with 30% polyethylene glycol 6000 (PEG 6000), 0.2 M sodium acetate pH 5, and 0.1 M calcium chloride. Crystals were harvested into cryo-protectant of 30% PEG 6000, 0.2 M sodium acetate, pH 5, 0.1 M calcium chloride, and 12% glycerol before vitrification in liquid nitrogen. X-ray diffraction data were collected at beamline I04 at Diamond Light Source (DLS). Crystals were of space group P2_1_2_1_2_1_ with cell dimensions of *a* = 53.06 Å, *b* = 90.33 Å, and *c* = 154.81 Å and diffracted to a resolution of 1.86 Å. Data were collected over 180° with a 0.2° Ω oscillation per image and 0.1-s exposure per image with an unattenuated 32- by 20-μm beam. Data were integrated using XDS and scaled using Aimless from the CCP4 suite (24–26). The structure of MtrC_C453A-sol_ was solved via molecular replacement using Phaser and MtrC (PDB accession no. 4LM8) as a search model. Alternating rounds of model building with COOT (27) and refinement using REFMAC5 were used to give a final model with an *R*_cryst_ (*R*_free_) of 15.1 (19.6%) (28) (Table S3).

10.1128/mbio.02589-22.3 TABLE S3 Data collection and refinement statistics for MtrC C453A (PDB accession no. 7QTH). Download Table S3, DOCX file, 0.01 MB. Copyright © 2023 Norman et al. 2023 Norman et al. https://creativecommons.org/licenses/by/4.0/ This content is distributed under the terms of the Creative Commons Attribution 4.0 International license.

### SEC-SAXS.

MtrC_sol_ and MtrC_C453Asol_ were concentrated to 7.5 mg/mL in 20 mM HEPES, pH 7.5, and 50 mM NaCl and analyzed on beamline B21 at DLS. Samples were passed through a Shodex KW402.5-4F column at 0.16 mL/min, and data were collected in the form of scans from the eluent. Scattered X-rays were measured using an Eiger 4M detector. Data were analyzed using ScÅtter IV (https://bl1231.als.lbl.gov/scatter/). Six to 10 scans with uniform radius of gyration (*R_g_*) values were averaged, and baseline was subtracted using baseline scans taken before the protein peak (Fig. S6).

Intensity of scattering factor Q curves (I(Q) curves) were analyzed using ATSAS software suite (29) to estimate the *R_g_* and Kratky plots to evaluate the overall compactness of the protein complexes. GNOM (30) was used to calculate the pair-distance distribution function [*P*(*r*)] to provide an independent estimation of *R_g_* and maximum dimension (*D*_max_). *Ab initio* bead modeling using DAMMIN generated molecular envelopes of MtrC_sol_ and MtrC_C453Asol_, aligned to the S. oneidensis MtrC crystal structure using SUPCOMB (31).

### Data availability.

The C453A MtrC structure and the associated structure factors are deposited in the Protein Data Bank under accession no. 7QTH. Data sets used to make figures are deposited at figshare (https://figshare.com/articles/dataset/Baseline_subtracted_scattering_curves_of_soluble_MtrC_and_MtrC_C453A_in_20_mM_HEPES_50_mM_NaCl_pH_7_5/21781943).
