# Supplementary material for: A Cysteine Pair Controls Flavin Reduction by Extracellular Cytochromes during Anoxic/Oxic Environmental Transitions
Source: mBio. 2023 Jan 16;14(1):e02589-22. doi: 10.1128/mbio.02589-22 (PMC9973256; doi:10.1128/mbio.02589-22)
Supplement: TABLE S1 [file mbio.02589-22-s0007.docx]

| Primer Name | Primer Sequence |
| --- | --- |
| MtrC_C444A_F | GTAGGTTGGTCAATGGCTTCTAGCGAAGGTAAG |
| MtrC_C444A_R | CTTACCTTCGCTAGAAGCCATTGACCAACCTAC |
| MtrC_C453A_F | TAAGTTTGTAGACGCTGCAGACCCTGCA |
| MtrC_C453A_R | TGCAGGGTCTGCAGCGTCTACAAACTTA |
| MtrC_C453S_F | CTAGCGAAGGTAAGTTTGTAGACTCTGCAGACCCTGCATTTGATGG |
| MtrC_C453S_R | CCATCAAATGCAGGGTCTGCAGAGTCTACAAACTTACCTTCGCTAG |
