# Supplementary figures and images for: A Cysteine Pair Controls Flavin Reduction by Extracellular Cytochromes during Anoxic/Oxic Environmental Transitions
Source: mBio. 2023 Jan 16;14(1):e02589-22. doi: 10.1128/mbio.02589-22 (PMC9973256; doi:10.1128/mbio.02589-22)

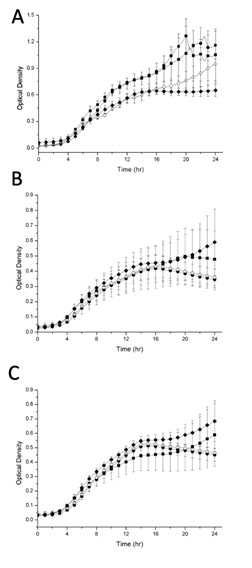

Supplement: FIG S1 [file mbio.02589-22-s0001.jpg]

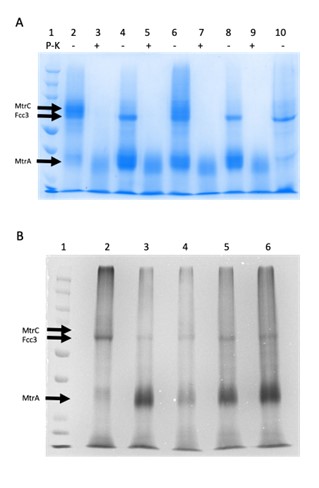

Supplement: FIG S2 [file mbio.02589-22-s0002.jpg]

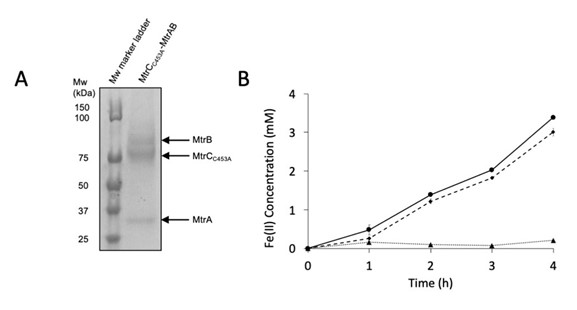

Supplement: FIG S3 [file mbio.02589-22-s0003.jpg]

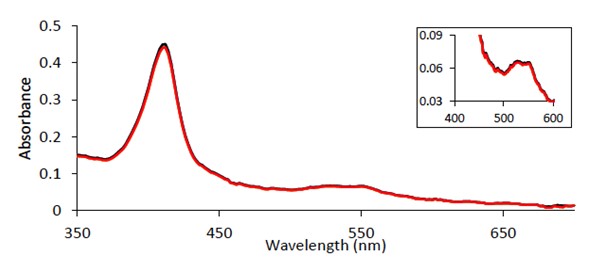

Supplement: FIG S4 [file mbio.02589-22-s0004.jpg]

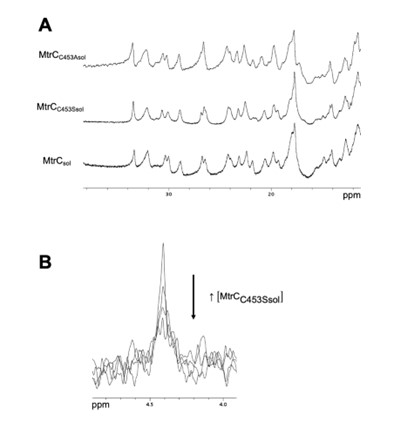

Supplement: FIG S5 [file mbio.02589-22-s0005.jpg]

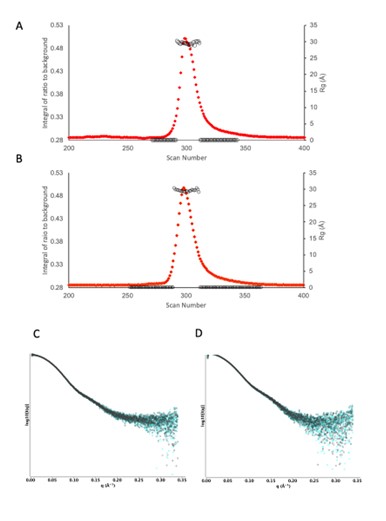

Supplement: FIG S6 [file mbio.02589-22-s0006.jpg]
