# Supplementary material for: A Cysteine Pair Controls Flavin Reduction by Extracellular Cytochromes during Anoxic/Oxic Environmental Transitions
Source: mBio. 2023 Jan 16;14(1):e02589-22. doi: 10.1128/mbio.02589-22 (PMC9973256; doi:10.1128/mbio.02589-22)
Supplement: TABLE S2 [file mbio.02589-22-s0008.docx]

| Plasmid Name | Vector | Tag | Membrane Anchor |
| --- | --- | --- | --- |
| pMtrC | pBAD202/D-TOPO | His | + |
| pC444A | pBAD202/D-TOPO | His | + |
| pC453A | pBAD202/D-TOPO | His | + |
| pC444A,C453A | pBAD202/D-TOPO | His | + |
| pMtrCsol | pBAD202/D-TOPO | Strep-II | - |
| pC453Asol | pBAD202/D-TOPO | Strep-II | - |
| pC453Ssol | pBAD202/D-TOPO | His | - |
