# Supplementary material for: A Cysteine Pair Controls Flavin Reduction by Extracellular Cytochromes during Anoxic/Oxic Environmental Transitions
Source: mBio. 2023 Jan 16;14(1):e02589-22. doi: 10.1128/mbio.02589-22 (PMC9973256; doi:10.1128/mbio.02589-22)
Supplement: TABLE S3 [file mbio.02589-22-s0009.docx]

|  | MtrC-C453A | |
| --- | --- | --- |
| **Data collection**  Wavelength | 0.97 Å | |
| Space group | P 2_1_ 2_1_ 2­_1_ | |
| Cell dimensions |  | |
| *a*, *b*, *c* (Å) | 53.06, 89.80, 154.29 | |
| α, β, γ (°) | 90.00, 90.00, 90.00 | |
| Resolution (Å) | 154.29-1.90 (1.94-1.90) | |
| *R*_merge_ (%)  *CC_1/2_* (%) | 9.0 (39.2)  99.7 (90.2) | |
| *I* / σ*I* | 14 (4) | |
| Completeness (%) | 99.5 (99.3) | |
| Multiplicity | 6.6 (5.9) | |
|  |  | |
|  |  | |
| **Refinement** |  | |
| Resolution (Å) | 1.90 | |
| No. reflections | 55739 | |
| *R*_work_ / *R*_free_ | 0.151/0.196 | |
| No. atoms |  | |
| Protein | 4787 | |
| Ligand/ion | 542 | |
| Water | 838 | |
| *B*-factors |  | |
| Protein | 17.9 | |
| Ligand/ion | 15.5 | |
| Water | 28.8 | |
| R.m.s. deviations |  | |
| Bond lengths (Å) | 0.015 | |
| Bond angles (°) | 2.8 | |
|  | |  |

*Values in parentheses are for highest-resolution shell.
